# Free energy simulations on a biomimetic glucose receptor: understanding the selectivity of GluHUT

**DOI:** 10.1039/d6sc00738d

**Published:** 2026-05-21

**Authors:** Ryan Eades, Marko Hanzevacki, Adrian J. Mulholland, Anthony P. Davis

**Affiliations:** a School of Chemistry, University of Bristol, Cantock's Close Bristol BS8 1TS UK Anthony.Davis@bristol.ac.uk; b Centre for Computational Chemistry, School of Chemistry, University of Bristol, Cantock's Close Bristol BS8 1TS UK Adrian.Mulholland@bristol.ac.uk

## Abstract

The bicyclic glucose receptor GluHUT represents a significant advance in supramolecular chemistry, binding glucose in water with an association constant of approximately 18 000 M^−1^ and exhibiting exceptional selectivity. Elucidating the molecular basis of this selectivity could be valuable for the rational design of receptors for other carbohydrate targets. In this study, we investigate the binding dynamics of GluHUT through extensive molecular dynamics simulations and enhanced sampling techniques, focusing on its complexation with glucose, galactose, and fructose in water. Umbrella sampling along a predefined reaction coordinate provides an atomistic view of the entire binding process, revealing the thermodynamic determinants of selectivity and the corresponding free energy profiles. Detailed analyses of hydrogen bonding networks, noncovalent interactions, and solvent reorganization demonstrate that even minor structural variations among sugars can lead to significant differences in binding affinity. These findings, together with the computed binding free energies that qualitatively agree with experimental trends, provide a mechanistic rationale for GluHUT's remarkable specificity toward glucose.

## Abstract 2

Molecular dynamics simulations on the synthetic carbohydrate receptor GluHUT throw light on the thermodynamics of binding and the selectivity towards glucose.

## Graphical abstract

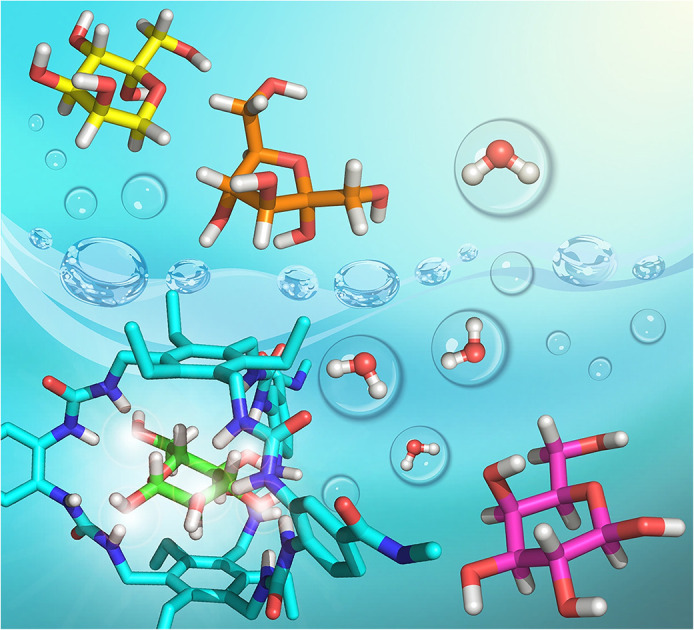

## Introduction

**Fig. 1:**
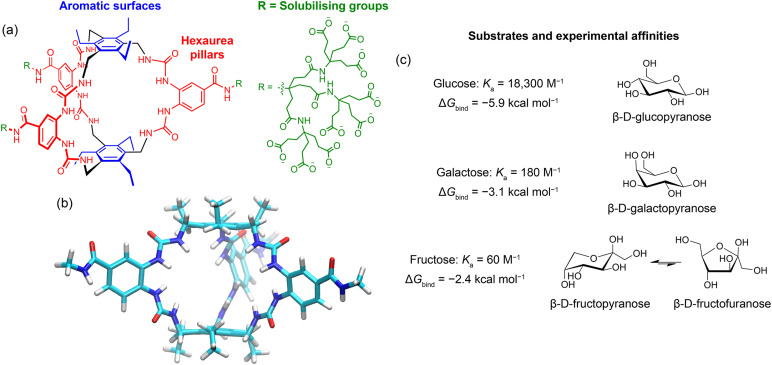
(a) Chemical structure of GluHUT. (b) Structure of the truncated GluHUT receptor model used in this work. (c) Carbohydrate substrate molecules studied in this work. The binding constant *K*_a_ values in water were obtained from Tromans *et al.*,^12^ and converted to binding free energies (Δ*G*_bind_) at 300 K. All three carbohydrates exist as mixtures of tautomers. For glucose and galactose, the major forms are the β-pyranose as shown. For fructose, both pyranose and furanose forms are significant; the major anomer is shown here for each.^15^

The selective binding of carbohydrates in aqueous solution presents a significant challenge. Saccharides are highly hydrophilic, interacting strongly with water, and share generally similar structures. A useful receptor must differentiate between subtly different targets, whilst also overcoming competition from solvent. Glucose is a ligand of particular biological importance, due to its central role in metabolism and, medically, its relevance to diabetes. Effective glucose receptors can lead to sensing systems which can help to advance the next generation of diabetes therapies.^1–3^ With nature as inspiration, our group in Bristol has synthesised a variety of carbohydrate receptors with a focus on glucose and other “all-equatorial” carbohydrates. The central design architecture has been christened a “temple”, with pillars rich in hydrogen bonding functionalities such as ureas or amides, combined with hydrophobic ‘roof’ and ‘floor’ units to accommodate the non-polar regions of the carbohydrates.^4–11^ The most recent, and successful, iteration of this architecture is GluHUT (Glucose binding HexaUrea Temple, Fig. 1a).^12^ With binding constants to glucose of *K*_a_ = 17 300–18 300 M^−1^ in phosphate buffered saline solution, GluHUT was a major breakthrough, surpassing previous systems by two orders of magnitude. It is also highly selective, favouring glucose over galactose by *a* factor of 100 and showing negligible affinities for non-carbohydrate targets. This meant that the molecule could be considered for use in medicine, suitable for both sensing and therapeutic applications. Its potential has recently been demonstrated in a glucose-responsive insulin which protects against hypoglycaemia in animal studies.^13^ Glucose sensitivity was achieved by attaching a GluHUT receptor to an insulin peptide at one end, and a β-glucoside to the other. Intramolecular binding dominates at low glucose concentrations, depressing insulin activity, whilst at higher concentrations the intramolecular interaction is overcome by glucose binding and insulin activity is restored.

Computational modelling was important in the design of GluHUT, but was limited to Monte Carlo molecular mechanics (MM) implemented through a standard commercial package.^14^ The calculations predicted an “all –NH in” conformation for the receptor core (Fig. 1a and b), and this was supported by 2D NMR experiments.^12^ It is understood that this well-defined structure is key to the binding properties of GluHUT, but detailed further investigation is needed to study the more intricate features that create such an ideal microenvironment for glucose, and also why other sugars bind much less strongly (see Fig. 1c for experimental binding affinities).^15^ Understanding why, for instance, galactose binds with 100-fold lower affinity than glucose, will help in designing the next generation of carbohydrate receptors targeting other biologically important ligands.

Host–guest binding has been investigated in various systems using a range of computational methods, including quantum mechanical (QM) calculations,^16–19^ MM Monte Carlo, and molecular dynamics (MD) simulations.^20–23^ A key advantage of MD simulations is the ability to combine them with enhanced sampling techniques in explicitly solvated environments, enabling the study of larger systems such as macromolecular complexes in water. Examples of such techniques include the adaptive string method, often applied to explore protein–ligand binding along the minimum free energy path,^24,25^ and metadynamics.^26,27^ In this work, we employed umbrella sampling, a path-based enhanced sampling method in which harmonic restraints are imposed across a series of overlapping windows along a predefined reaction coordinate, to obtain the potential of mean force (PMF), from which binding free energies were derived.^28–31^ This technique provides transparent and well-controlled convergence for one-dimensional collective variables (such as centre of mass distances), making it particularly suitable for studying binding and unbinding events of small-molecule guests. Umbrella sampling has been successfully applied to both relatively simple host–guest systems such as β-cyclodextrin,^29,32–34^ and more complex biomolecules.^31,35,36^ We found that the relative computed binding free energies qualitatively reproduced the experimental trends for the monosaccharides studied in this work. Analysis of the trajectories shows that hydrogen bonding and solvent effects explain the origins of the binding selectivity. The results suggest that these methods can be used to inform the design of new receptors aimed at other targets of biological importance.

## Methods

### System preparation

An open-cage structure of GluHUT was built in Avogadro 1.2.0,^37^ with all urea protons pointing into the cavity, as suggested by 2D NMR data.^12^ The structure was truncated at the outer amide linker, replacing the polycarboxylate dendrimer with a methyl group to allow for smaller simulation boxes and thus more extensive sampling (Fig. 1b). The resulting structure was first energy-minimised in Avogadro 1.2.0 using the general amber force field (GAFF).^38^ This structure was then optimised at the QM level using the B3LYP/6-31G(d) method in Gaussian 16.^39,40^ Partial atomic charges for the host were obtained using a standard RESP fitting procedure based on ESP calculations at the HF/6–31G(d) level of theory.^41–43^ All bonded and nonbonded parameters for GluHUT were described using GAFF.^38^ Force field parameters for carbohydrate guests (glucose, galactose, and fructose) were taken from GLYCAM06.^44^

### Molecular dynamics simulations

The host–guest complexes of GluHUT with the three sugars were explored using all-atom molecular dynamics (MD) simulations. The TIP3P water model was used to simulate explicit water.^45,46^ For comparison, the TIP4P water model was also tested yielding very similar results but at higher computational cost. The solute (empty host and host–guest complex) was placed in a 15 Å octahedral box containing ≈4000 water molecules, giving an approximate simulation concentration of 14 mm All MD simulations were performed with the *pmemd.cuda* module of AMBER 24 program,^47,48^ under periodic boundary conditions (PBC) in all directions. Long-range electrostatic interactions were treated with the particle mesh Ewald (PME) method using a real-space cutoff of 10 Å, which was also applied for Lennard-Jones interactions.

Each system was minimised for 5000 steps, including 250 steepest descent steps and the remaining steps using the conjugate gradient algorithm. The systems were then equilibrated by gradual heating from 100 K to 300 K under NVT conditions during 2 ns, followed by density equilibration under NPT ensemble for 2 ns at 300 K. The temperature was controlled using Langevin dynamics with a collision frequency of 1.0 ps^−1^, and a time step of 2 fs was used. Bonds involving hydrogen atoms were constrained using the SHAKE algorithm, and all production simulations were carried out under NPT conditions.^49^ For each system, three independent production runs of 100 ns each were performed, yielding a total of 300 ns of unrestrained MD per system.

### Steered and umbrella sampling simulations

**Fig. 2:**
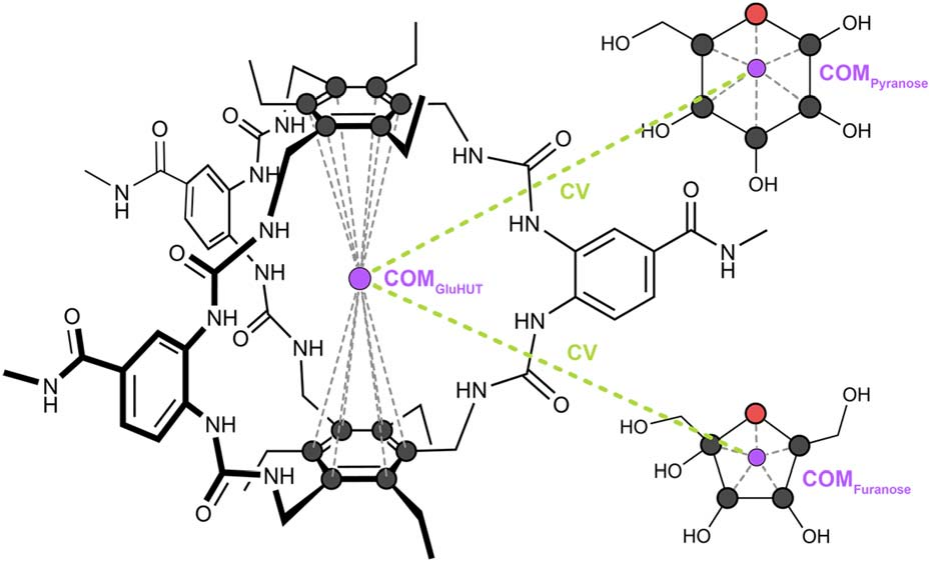
Graphical representation of the collective variable (CV) used as the reaction coordinate in this work. The atoms used to define the centres of mass (COM) of the GluHUT host, and the carbohydrate guest are shown as black (carbon) and red (oxygen) circles, respectively, with each COM indicated by a purple circle. The CV is defined as the distance between the host and guest COMs, illustrated by the green dashed line.

Initial host–guest unbinding trajectories were generated using steered molecular dynamics (SMD) simulations.^50,51^ The starting structures for these simulations were the final snapshots from the unrestrained MD simulations of each sugar complexed with GluHUT. For each guest, we performed five independent SMD simulations, using the reaction coordinate (RC) as the distance between the centre of mass (COM) of the guest ring atoms and the COM of the host cavity (defined as the COM of twelve carbon atoms from the benzene “roof” and “floor” units). See Fig. 2 for details. Unbinding was induced by increasing this distance from 0 to 15 Å over 30 ns with a harmonic restraint of 200 kcal mol^−1^ Å^−2^. Due to a lack of convergence within these SMD simulations, conclusions regarding free energies were not drawn. Their use therefore was to provide diverse structures across the reaction coordinate, including multiple sugar exit paths from the cavity.

Potentials of mean force (PMFs) for guest binding to GluHUT were calculated using umbrella sampling simulations along the predefined reaction coordinate. Three SMD trajectories were used per sugar as starting points for the umbrella sampling simulations. The choice of these trajectories was based on sampling different pathways that the sugar exited the cavity (three main routes possible). For the pyranose tautomers, two main pathways were observed. In path A, the sugar ring oxygen atoms exited the cavity first. In path B, the sugar exited the cavity leading with the CH_2_OH group. Each trajectory yielded a total of 51 umbrella windows with *a* spacing of 0.3 Å and a force constant of 50 kcal mol^−1^ Å^−2^. Each window was subjected to 10 ps relaxation keeping the restraints and then simulated for 30 ns under NVT conditions at 300 K. PMFs were reconstructed at 300 K using the weighted histogram analysis method (WHAM) with 400 bins per window (Fig. S1).^52,53^ Binding free energies were determined from each PMF as the difference between the global free energy minimum (bound basin) and the mean free energy in the bulk region (RC ≈ 13–15 Å). Reported values represent the mean binding free energy ± standard deviation from three independent PMFs per sugar. The time evolution of the reaction coordinate during the initial unrestrained simulations was monitored for each species, and the resulting RC values corresponded closely to the global PMF minima (Fig. S2). The two types of pathways produced similar PMFs, except for galactose where the shape of the free energy landscape differed significantly across the reaction coordinate (Fig. S3).

### Radial distribution function

To investigate the solvent distribution around the solutes, radial distribution functions (RDFs) and average solvent occupancy were computed. RDFs of water oxygen atoms were calculated from each urea proton. To compute the number of water molecules in the cavity, we used the watershell option available in *cpptraj*, which counts the per-frame number of water molecules in the first solvation shell based on the distance criteria. The cutoff for the first water shell was determined from the first minimum in the RDF at 2.65 Å. The average numbers of water molecules within this distance for empty and complexed GluHUT, taken from three 100 ns unrestrained simulations, were then reported as water occupancy. Uncertainties were reported as standard errors of the mean, from the three replicates.

### Grid inhomogeneous solvation theory

For more detailed insight into water thermodynamics, grid inhomogeneous solvation theory (GIST)^54,55^ was employed to compute spatially resolved water properties around the empty GluHUT receptor. GIST divides space around the receptor into 3D grid (0.5 Å spacing was used here) and calculates water density, energy and entropy for water molecules in each grid cell relative to bulk. Similar analyses have been previously carried out to study host–guest complexes of cyclodextrins and cucurbit[*n*]urils.^56^ The plotted quantity here is the total normalised entropy per water molecule, −*T*Δ*S*_tot_ = −*T*(Δ*S*_trans_ + Δ*S*_ori_) in kcal mol^−1^ per water, where negative values indicate water more ordered than bulk. Regions of low water entropy indicate sites where water molecules are more ordered than in bulk solvent, contributing an unfavourable free energy penalty. Displacement of these ordered waters by sugar binding provides a thermodynamic driving force for host–guest complexation.

The empty GluHUT receptor in a large water box (≈25 000 water molecules) was first equilibrated for 2 ns of NPT simulation, followed by a 100 ns unrestrained NVT production simulation. A representative structure was extracted from the free energy minimum, identified *via* principal component analysis of all nonhydrogen GluHUT atoms. This structure was further equilibrated by 2 ns of NVT simulation with harmonic positional restraints (100 kcal mol^−1^ Å^−2^) on the heavy atoms of GluHUT, followed by 50 ns of restrained NVT simulation. From this trajectory, 2500 snapshots were processed using the GIST analysis in the *cpptraj* program.^57^ The key outputs are the translational (Δ*S*_trans_) and orientational (Δ*S*_ori_) water entropies per water molecule relative to bulk, reported as normalised entropy terms. The main limitations of GIST are that it relies on classical force fields and assumes water molecules are independent within each grid cell, potentially underestimating water–water cooperativity. It provides per-water properties rather than absolute energies.

### Linear interaction energy

Electrostatic and van der Waals contributions to binding were investigated using the linear interaction energy (LIE) method across umbrella sampling windows spaced at 0.3 Å along the RC from 0 Å to 15 Å, using the *cpptraj* module of AMBER 24.^57^

### Unrestrained simulations of the bound states

To sample distinct binding orientations, three additional 100 ns unrestrained MD simulations, equilibrated using the same protocol as described above, were performed for each complex. Starting structures were selected from the initial unrestrained simulations and rotated about the *z*-axis relative to the host COM (rotation angles: 0°, 120° and 240°). This created three unique binding orientations which enabled sampling of a more diverse set of complexes.

### Hydrogen bonds analysis

Hydrogen bonds were analysed using *cpptraj*, defined by the following criteria: –NH and –OH groups could act as donors, while C=O and C–OH groups could act as acceptors. The donor–acceptor distance cutoff was 3.0 Å, with a minimum angle of 150° for directionality. For hydrogen bonds involving water molecules, a more permissive angle cutoff of 135° was used to better capture their transient nature.

Hydrogen bonding analysis revealed that the sugar hydroxyl oxygens acted exclusively as acceptors with the receptor, whilst they served primarily as acceptors and occasionally as donors with water molecules.

### Noncovalent interaction

Noncovalent interaction (NCI) analysis was performed using the independent gradient model (IGM),^58^ a real-space method that identifies and quantifies noncovalent interactions by analysing gradients in electron density. This analysis was based on promolecular densities derived from 100 representative MD snapshots of glucose and galactose bound to GluHUT. The sign(*λ*_2_)*ρ* function was visualised as blue-green-red isosurfaces (isovalue = 0.02 a.u.), with blue indicating strong attractive interactions, green denoting weaker noncovalent interactions and red representing steric clashes. IGM analysis was carried out using the Multiwfn program.^59^ All structures were visualised in VMD,^60^ and plots were generated with Matplotlib.^61^

## Results and discussion

### Selectivity of GluHUT for binding glucose reproduced with free energy simulations

To study GluHUT's binding properties, four different guest molecules were investigated in this work: β-d-glucose, β-d-galactose, β-d-fructopyranose, and β-d-fructofuranose (further referred to as glucose, galactose, fructopyranose, and fructofuranose). For glucose and galactose, the tautomers chosen for study were the β-pyranose forms, which predominate in aqueous solution (Fig. 1c). In the case of glucose there is evidence that the β-pyranose is the major substrate for GluHUT, and it seems likely that the same is true for galactose.^12^ In the case of fructose, the form which is bound is less certain as both furanose and pyranose tautomers are present in water. We therefore chose to study both, as the major (β) anomers (Fig. 1c).^15^

Prior to umbrella sampling, a series of steered MD (SMD) simulations of the unbinding process of each sugar from the receptor were carried out. Starting with a bound conformation, these simulations involved ‘pulling’ the monosaccharide out of the GluHUT host at a constant speed. Due to the high pulling speed, convergence in the free energy (or work) was not obtained and no differentiation between sugars was observed in SMD simulations. The primary use of these trajectories was to generate snapshots for subsequent umbrella sampling simulations.

**Fig. 3:**
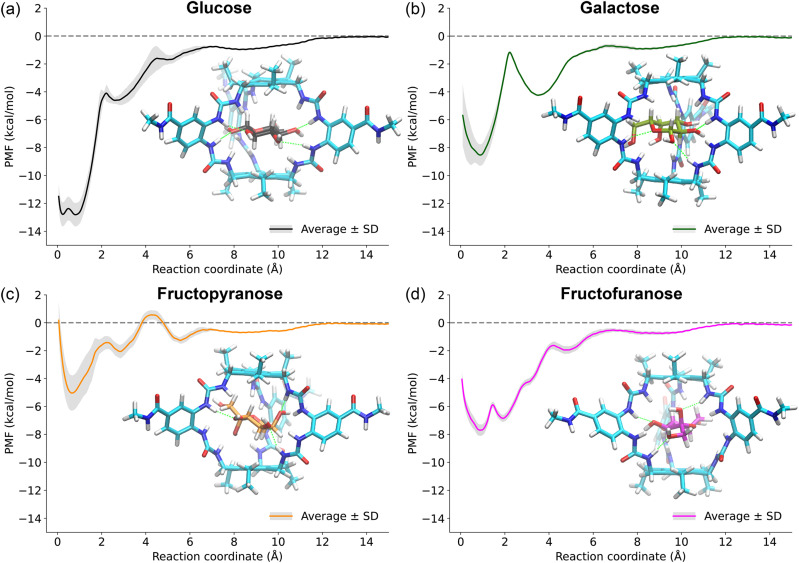
The average potential of mean force (PMF) for (a) glucose, (b) galactose, (c) fructopyranose, and (d) fructofuranose binding to GluHUT obtained from three independent umbrella sampling simulations at 300 K, along with a representative structure of the bound state. Shaded regions correspond to errors across the PMF, determined by the standard deviation of the three independent PMFs per sugar. See Fig. S1 for independent PMFs used to construct average PMFs. The binding free energies are calculated by subtracting the energy of the global minimum in the PMF from the energy in the bulk. For clarity, only PMFs for galactose path B were reported here, with path A also possible as shown in Fig. S3 and S4. The simulation concentration was 14 mM and concentration corrected affinities were calculated using eqn (S1): glucose = −10.0 kcal mol^−1^, galactose = −5.6 kcal mol^−1^, fructofuranose = −5.2 kcal mol^−1^, and fructopyranose = −2.5 kcal mol^−1^.

The potentials of mean force (PMFs) obtained from these simulations show qualitative agreement with the trends in the experimentally derived binding free energy values (Fig. 3 and Table 1). The computed binding free energy for glucose was −12.5 ± 0.9 kcal mol^−1^, compared to the experimental value of −5.9 kcal mol^−1^. For galactose, the binding free energy along path B was calculated to be −8.1 ± 1.9 kcal mol^−1^, in comparison to the experimental value of −3.1 kcal mol^−1^. Notably, the alternative path A for galactose yields a slightly weaker binding affinity of −6.9 ± 0.3 kcal mol^−1^.

**Table 1:** Experimental, computed, and concentration-corrected binding free energies (Δ*G*_bind_) for each sugar bound to GluHUT. Values are given in kcal mol^−1^

Sugar	Experimental	Computed	Conc. corrected
Glucose	−5.9	−12.5 ± 0.9	−10.0
Galactose	−3.1	−6.9 ± 0.3 (path A)	−4.4
		−8.1 ± 1.9 (path B)	−5.6
Fructopyranose	−2.4	−5.0 ± 1.3	−2.5
Fructofuranose		−7.7 ± 0.5	−5.2

For fructose, fructopyranose interacts relatively weakly with the GluHUT cavity, exhibiting a computed binding free energy of −5.0 ± 1.3 kcal mol^−1^. It also shows greater mobility within the cavity compared to glucose or galactose, consistent with its weaker affinity. In contrast, umbrella sampling simulations for fructofuranose yielded a significantly more negative binding free energy of −7.7 ± 0.5 kcal mol^−1^. These results suggest that fructofuranose, although present only as 20% of the tautomeric mixture at equilibrium, might be the predominant binding form of fructose with GluHUT in solution.

### PMFs reveal multiple minima

The galactose PMF exhibited a pronounced local free energy minimum at RC ≈ 3.9 Å, observed only along unbinding path B (CH_2_OH exits first; Fig. S3). This metastable state was absent in path A (ring oxygens exit first), while glucose PMFs showed no path dependence, yielding more similar free energy profiles. To study the stability of this metastable state, extensive unrestrained MD simulations starting from RC = 3.9 Å were carried out using the same conditions as the simulations of the bound states. These simulations confirmed path B's relative stability for galactose: two replicates returned to the global bound minimum, while one persisted in the metastable state; path A structures collapsed immediately, indicating no true minimum.

Despite similar PMF values for glucose and galactose at RC ≈ 3–4 Å, this path dependence is unique to galactose. We therefore compared structures of both sugars in this RC range. Representative snapshots are shown in Fig. S4. Glucose forms multiple hydrogen bonds with GluHUT in both paths, adopting a parallel orientation relative to the receptor's “temples”. Galactose path B achieves similar ring-oxygen hydrogen bonding. However, path A requires substantial sugar rotation due to steric clash from the axial C_4_–OH group, preventing stable hydrogen bonding and eliminating the local minimum.

The PMF of fructofuranose revealed two clearly defined bound states, within 1 kcal mol^−1^. The lowest energy state was at RC = 0.9 Å, with the other minimum at RC = 2.1 Å. From the time evolution of the reaction coordinate shown in Fig. S5, it was found these states interconverted within the duration of unrestrained simulations.

Experimental kinetic data are not available. We do not draw conclusions regarding the binding kinetics here, which would require more consideration of dynamics of binding. Instead, our analysis focuses on the free energy profiles along the predefined reaction coordinate, and on how the trends in the calculated minima compare with the experimentally measured binding affinities. Our study focuses on equilibrium binding affinities derived from PMF profiles rather than kinetic rates. It would be interesting to analyse binding kinetics in detail which is possible through simulation.^62^ That study also investigates electronic polarisation changes during binding through multiscale QM/MM simulations, an effect not included in standard MM MD simulations. The systematic overestimation of the calculated binding free energies (Δ*G*_bind_) relative to experiment may be in part due to the use of a standard non-polarisable force field, which neglects electronic polarisation changes.

To understand the origin of the differences in affinity, the unrestrained simulations of the complex at the minimum of the PMF were first analysed using the linear interaction energy (LIE) method.^63^ This showed that electrostatic interactions are the dominant driver for binding of all four sugars, with glucose having the largest contribution of −38 ± 5 kcal mol^−1^ in the bound state, followed by galactose at −33 ± 6 kcal mol^−1^, fructofuranose at −30 ± 5 kcal mol^−1^, and fructopyranose at −27 ± 5 kcal mol^−1^. Details of the LIE analysis across the entire unbinding coordinate are shown in Fig. S6. The bound states were further analysed through computing water occupancy in the cavity and hydrogen bonding (H-bonding) interactions for receptor–ligand, receptor–water, and ligand–water.

### GIST uncovers entropy cost of bound waters in GluHUT

**Fig. 4:**
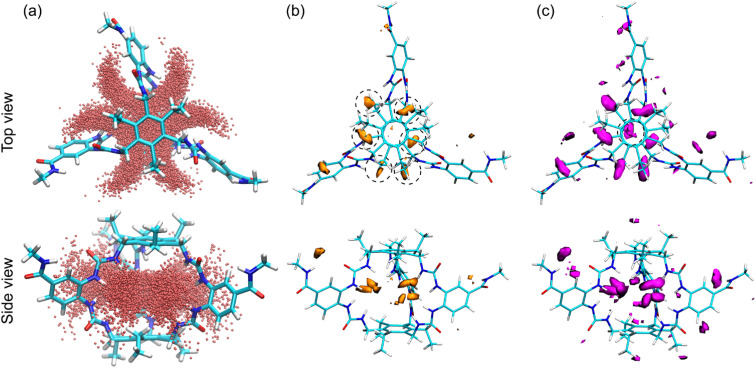
(a) Top and side views of the water occupancy in the empty receptor. (b) GIST isosurfaces showing the total entropic contribution to hydration free energy as a sum of translational and orientational entropic contributions derived from water positional and angular distributions. Isosurfaces represent water sites at two levels of negative (unfavourable) entropy, (b) the orange surfaces being more negative (−0.1 kcal per mol per water) than the (c) magenta surfaces (−0.06 kcal per mol per water). Both sets of surfaces are shown from two perspectives.

Before considering the effects of water on receptor–ligand complexes, we first examined the empty receptor. Operating in water, GluHUT's binding energetics are profoundly influenced by solvent reorganisation, where cavity-resident water molecules act both as competitors and thermodynamic modulators.^64,65^ Number density calculations on unrestrained simulations of the empty receptor revealed that the cavity maintains an average hydration shell of seven water molecules, predominantly arranged in “horseshoe-shaped” distributions around the urea –NH groups (Fig. 4a). These tightly bound waters accept hydrogen bonds (total three on average) from the urea motifs and avoid hydrophobic roof/floor regions. Displacement of these ordered waters during monosaccharide binding provides potential entropy gains, as shown by grid inhomogeneous solvation theory (GIST).^66^

The total entropy (Δ*S*_tot_) contribution to the hydration free energy of the receptor derived from GIST analysis quantifies the first-order entropic penalty experienced by water molecules in each voxel of the grid, encompassing both translational (Δ*S*_trans_) and orientational (Δ*S*_ori_) components. These values (Fig. 4), measure how much the ordering of water inside GluHUT deviates from bulk behaviour, reflecting the loss of configurational freedom when water molecules interact with the host. A large negative entropic penalty signifies that water in that region is more ordered and constrained than in bulk, possessing fewer accessible microstates. In contrast, positive or less negative terms indicate water with greater freedom, behaving more similarly to bulk solvent. Through this analysis, regions in the host cavity with highly negative total water entropy can be identified as sites where water molecules are likely to be favourably displaced upon guest binding. The analysis reveals six regions of low-entropy water within the cavity, paired with the six urea groups in GluHUT and reflecting H-bonding to the urea –NHs (Fig. 4b, orange and magenta isosurfaces). A seventh region in the centre of the cavity appears at the less negative level (magenta isosurface). Displacement of these ordered waters by a single, relatively rigid carbohydrate molecule would substantially increase the system's entropy, making such replacement thermodynamically favourable from an entropic perspective.

### Glucose expels water from GluHUT's cavity

**Fig. 5:**
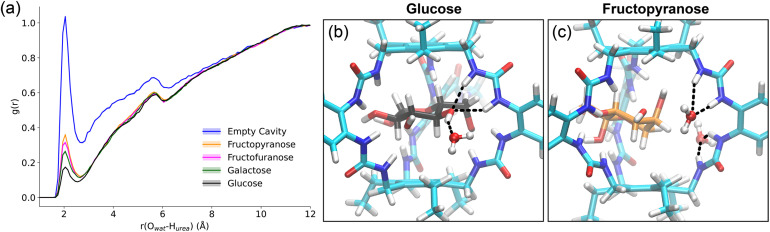
(a) The radial distribution function (RDF) of water oxygen atoms calculated from each of the urea protons (averaged) within GluHUT's cavity, for each of the four sugar complexes, and the empty cavity. (b) Representative glucose complex structure showing glucose in a central binding orientation within the cavity, receiving hydrogen bonds from a urea whilst donating a H-bond to a nearby water molecule. (c) Representative binding orientation of fructopyranose showing two water molecules competing for the urea binding sites.

Radial distribution function (RDF) analysis of the complexes, and the empty receptor, showed that glucose achieved significant dehydration of the cavity, with galactose having slightly more water present. Both tautomers of fructose permitted some water occupancy (≈2.5 molecules) in the cavity (Fig. 5a and Table S1). Whilst RDFs give an insight into the probability of finding water in the cavity, measuring the number of H-bonds can help to quantify this. The H-bond frequency between water oxygens and urea protons revealed an average 0.46 H-bonds between the cavity and water when glucose was bound, whilst this number was 0.78 for galactose and 0.91 for fructofuranose. Fructopyranose had the most competition with water, at 0.97 H-bonds formed (see Table S2 for uncertainties, which are small). This can be visualised by an increased density of water at the edge of the cavity interacting with two adjacent urea groups, competing for H-bonds with the sugar (Fig. 5b and c).

### Host–guest hydrogen bonding dominates selectivity

**Fig. 6:**
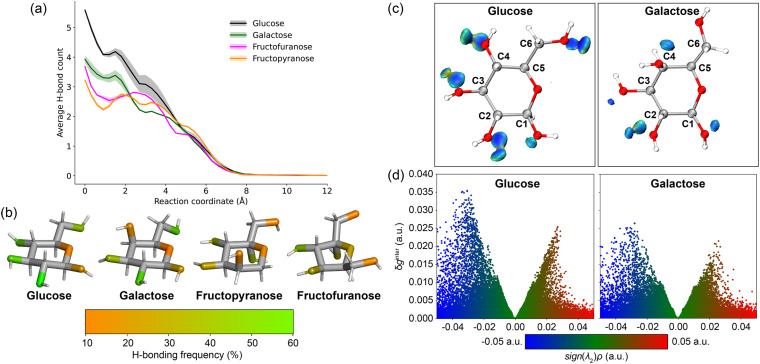
(a) H-bonding between GluHUT and each guest, along the binding reaction coordinate, averaged from 3 umbrella sampling runs. Note at RC = 0 Å, the hydrogen bonding is significantly higher for all sugars than at their bound basin. Due to restraints applied during umbrella sampling, complexes which have more hydrogen bonds with the receptor exist, but these are not characterised at the global free energy minimum. (b) Colour-coded H-bond frequency scores for each of the sugar oxygens (as H-bond acceptors) with GluHUT. (c) Sign(*λ*_2_)*ρ* coloured isosurfaces of *δg*_inter_ = 0.02 a.u. from independent gradient model (IGM) analyses of glucose and galactose with GluHUT. The blue isosurfaces indicate strong attractive interactions, such as H-bonds, green isosurfaces indicate weaker noncovalent interactions such as van der Waals, whilst red indicates repulsive interactions, such as steric clashes. Only the carbohydrate guest is shown for clarity. (d) IGM scatter plots showing sign(*λ*_2_)*ρ* against *δ*g_inter_ descriptor for glucose and galactose in GluHUT.

Based on the original modelling of GluHUT, H-bonding between host and guest is expected to be a key determinant of binding selectivity.^12^ Given that our simulations are fully solvated, the analysis of hydrogen bonding of these systems need not be limited to host–guest interactions, but also guest-solvent and host-solvent. This in totality can help rationalise the phenomena we see here, highlighting the origins of selectivity for glucose. To start, host–guest H-bonds were characterised across the entire PMF, using umbrella windows between RC = 0 Å and 15 Å. The results depicted in Fig. 6a show that glucose has the most H-bonding with the receptor in the bound state, averaging 4.1 H-bonds between the sugar oxygens and the urea –NH atoms. Galactose formed 3.1 H-bonds, with fructofuranose having 3.2, and finally fructopyranose forming only 2.3 H-bonds in the bound state. See Fig. S7 and Table S3 for more details.

Clearly, the structural differences between the four sugars are the key to the differences in binding free energy. Our results indicate that H-bonding is the predominant driver for binding. Therefore, additional unrestrained simulations of the bound complexes were performed (see Methods for details). The H-bonding between each sugar oxygen and the receptor was characterised, and scores were assigned to each oxygen, based on the frequency of H-bonds with GluHUT. Colour-coded representations of each sugar show which oxygens interacted most with the receptor, with orange and green indicating lower and higher percentages of interaction, respectively (Fig. 6b). Fructopyranose, which has a more globular structure than the other sugars, showed relatively low H-bonding: none of the oxygens show strong H-bonding, suggesting a mismatch with the cavity. Fructofuranose showed relatively strong H-bonding to the oxygens directly attached to its furanose ring, while the CH_2_OH groups interacted less frequently. This may account for the tighter complex, given these groups are more flexible and less sterically hindered within the cavity, than for the bulky fructopyranose (see Table S4).

The most interesting comparison is between glucose and galactose, given their structures only vary by the stereochemistry of –OH in the C4 position (see Fig. 6c). The axial –OH of galactose shows a relatively low H-bond frequency with the receptor, whilst the equatorial –OH of C4 for glucose frequently engaged in H-bonding. Noncovalent interaction (NCI) analysis revealed fine differences in the H-bonding of glucose and galactose to GluHUT (Fig. 6c).^58,59^ Namely, IGM isosurfaces confirmed strong H-bonding of the C4–OH of glucose with GluHUT. In contrast, for galactose this interaction of the axial C4–OH was much less prevalent. The IGM scatter plots depicted more negative (blue) values indicating a larger number of attractive noncovalent interactions (specifically H-bonds) for glucose compared to galactose.

### Insights from sugar–water interactions

Host–guest hydrogen bonds are clearly important in describing selectivity, but interactions with water must also be considered. Therefore, per-oxygen H-bond mapping approach was carried out for both glucose and galactose, where water acted as both the donor and the acceptor. It was found that the sugar oxygens were largely H-bond acceptors in the bound state, rarely donating to water. From unrestrained simulations, glucose formed ∼4 hydrogen bonds with water compared to ∼3 for galactose. Furthermore, the axial C4–OH of galactose formed almost no H-bonds with water, whilst the corresponding –OH for glucose was 0.7 on average (see Table S5 for details). These enhanced interactions with solvent enable glucose to maintain stronger simultaneous H-bond networks with both receptor and solvent, thereby amplifying selectivity.

## Conclusions

Our simulations of GluHUT's sugar binding mechanisms reveal the molecular basis of glucose recognition and specificity. Molecular dynamics simulations reproduce the remarkable selectivity of this receptor, helping to provide a comprehensive understanding of how subtle structural differences dramatically influence binding interactions. They show four critical aspects of GluHUT's binding behaviour. First, enhanced sampling relative binding free energy simulations qualitatively reproduced experimental trends in binding affinities, distinguishing between strong binders like glucose and weaker binders like galactose and fructose. Radial distribution function analysis, supported by grid inhomogeneous solvation theory, revealed the important role of water displacement in the binding process. The role of water in stabilizing complexes through hydrogen bonding was also revealed, particularly in the case of glucose where there seems to be a complementary effect of water, providing around 4 hydrogen bonds to the bound guest. And finally, hydrogen bonding between the host and the guest is the predominant driving force for complex formation. Glucose forms 4 hydrogen bonds with the receptor, while galactose forms one hydrogen bond less. These findings not only reproduce experimental selectivity trends but also, through detailed analyses of hydrogen bonding, noncovalent interactions, and solvation effects, reveal the subtle mechanisms driving GluHUT's profound selectivity for glucose.

In the longer term, these studies may serve as a basis for predicting the behaviour of new receptor designs. The lack of reliable methods for assessing designs presents a major obstacle to progress in biomimetic carbohydrate recognition, and to work in related areas. Standard desktop modelling packages have proved of limited use, probably because they rely on continuum solvation and estimate energies rather than free energies. Methods based on MM MD could provide a way forward, greatly extending the scope of receptor design.

## Author contributions

R. E.: methodology, data curation, formal analysis, investigation, visualization, writing of the original draft, review and editing. M. H.: methodology, supervision, conceptualization, review and editing, formal analysis and visualization. A. J. M.: conceptualization, supervision, review and editing, funding acquisition. A. P. D.: conceptualization, supervision, review and editing, funding acquisition.

## Conflicts of interest

There are no conflicts to declare.

## Supplementary Material

SC-OLF-D6SC00738D-s001

## Data Availability

The SI Fig. S1–S8 and Tables S1–S5 can be found in the supplementary information (SI). Simulation data is available on Zenodo: https://doi.org/10.5281/zenodo.19664426. Supplementary information is available. See DOI: https://doi.org/10.1039/d6sc00738d.
