# Supplementary material for: Free energy simulations on a biomimetic glucose receptor: understanding the selectivity of GluHUT
Source: Chem Sci. 2026 May 21;17(22):10896–906. doi: 10.1039/d6sc00738d (PMC13215116; doi:10.1039/d6sc00738d)
Supplement: SC-017-D6SC00738D-s001 [file SC-017-D6SC00738D-s001.pdf]

Supplementary Information (SI) for Chemical Science.
This journal is © The Royal Society of Chemistry 2026

Supplementary Information

Free Energy Simulations on a Biomimetic Glucose Receptor: Understanding the Selectivity of GluHUT

Ryan Eades,[a] Marko Hanzevacki,[b] Adrian J. Mulholland,[*,b] Anthony P. Davis[*,a]

[a] School of Chemistry, University of Bristol, Bristol, United Kingdom

[b] Centre for Computational Chemistry, School of Chemistry, University of Bristol, Bristol, United Kingdom

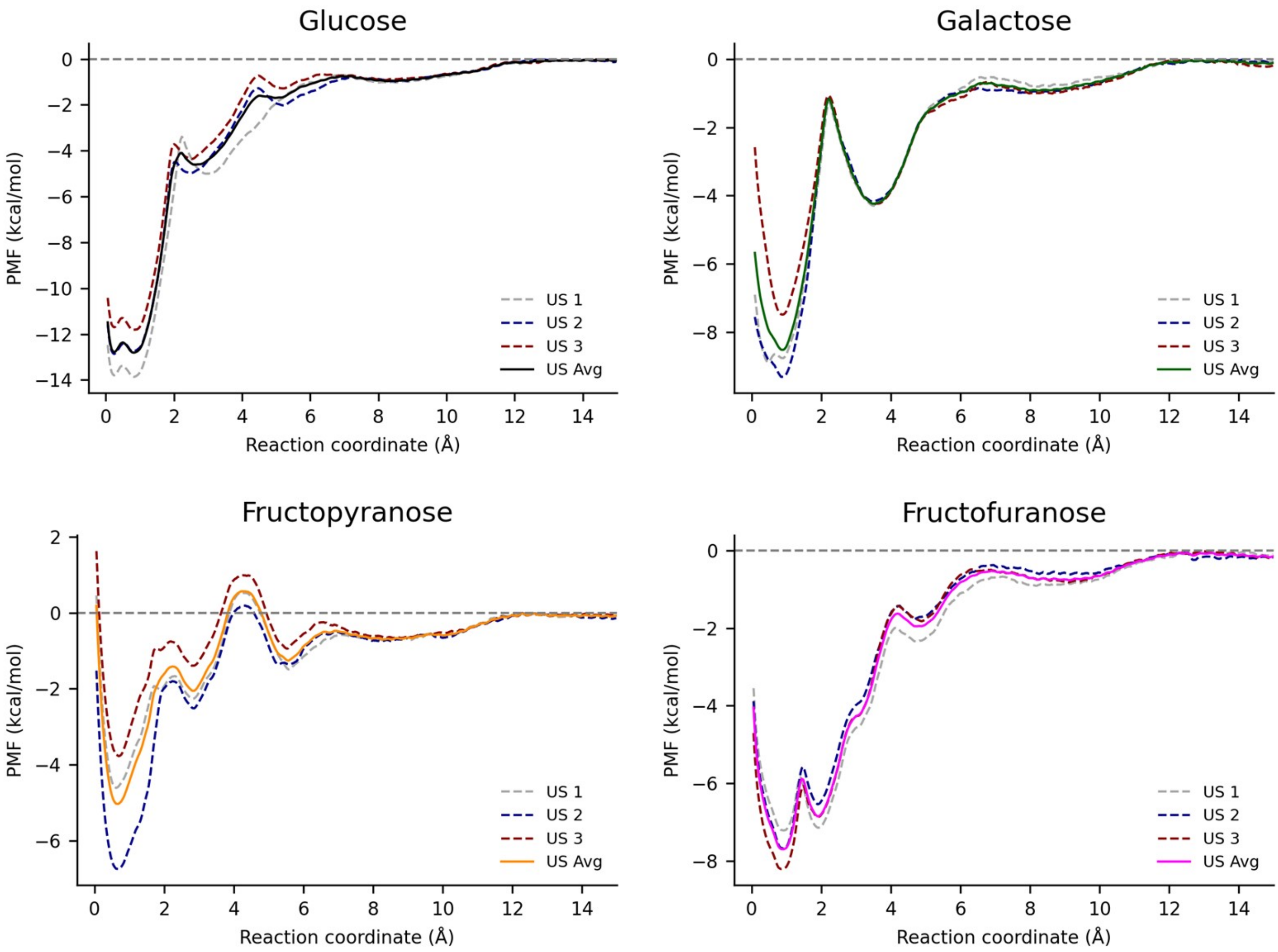

Figure S1. PMFs from umbrella sampling molecular dynamics simulations. Potentials of mean force (PMFs) derived using WHAM from umbrella sampling (US) simulations for each of the four guest molecules. The standard deviations were determined from the three umbrella sampling runs, taking the energy gap between the global minimum at RC ≈ 0.5 Å and the average value in the bulk at RC > 13.0 Å.

$$\Delta G^o = \Delta G_{Sim} + RT ln\left(\frac{C}{C^o}\right)$$

Equation S1. The concentration corrected Gibbs free energy equation.

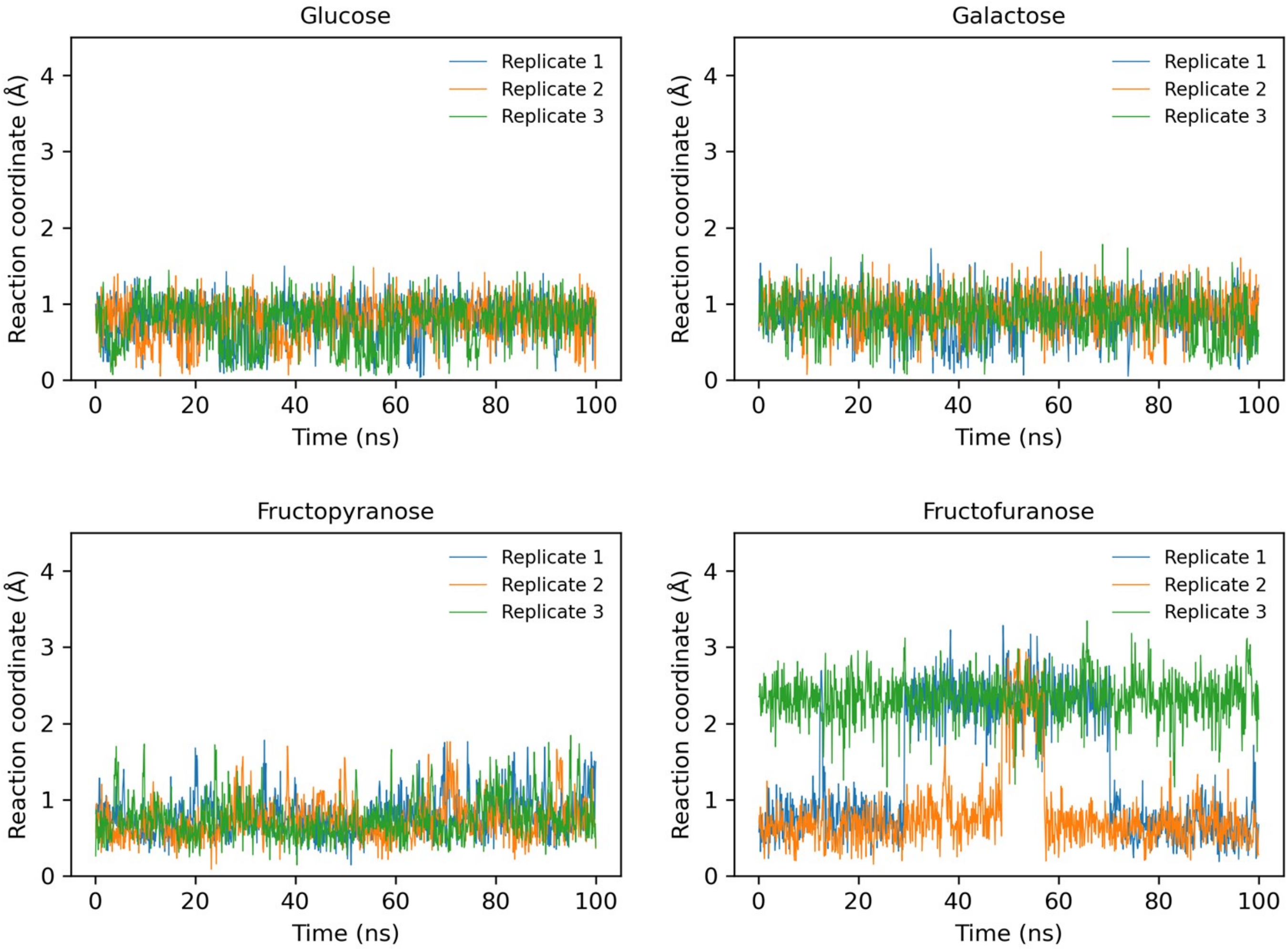

Figure S2. Unrestrained molecular dynamics simulations of bound states. Time evolution plots of the reaction coordinate obtained from three replicates of 100 ns unrestrained MD simulations of each host-guest complex.

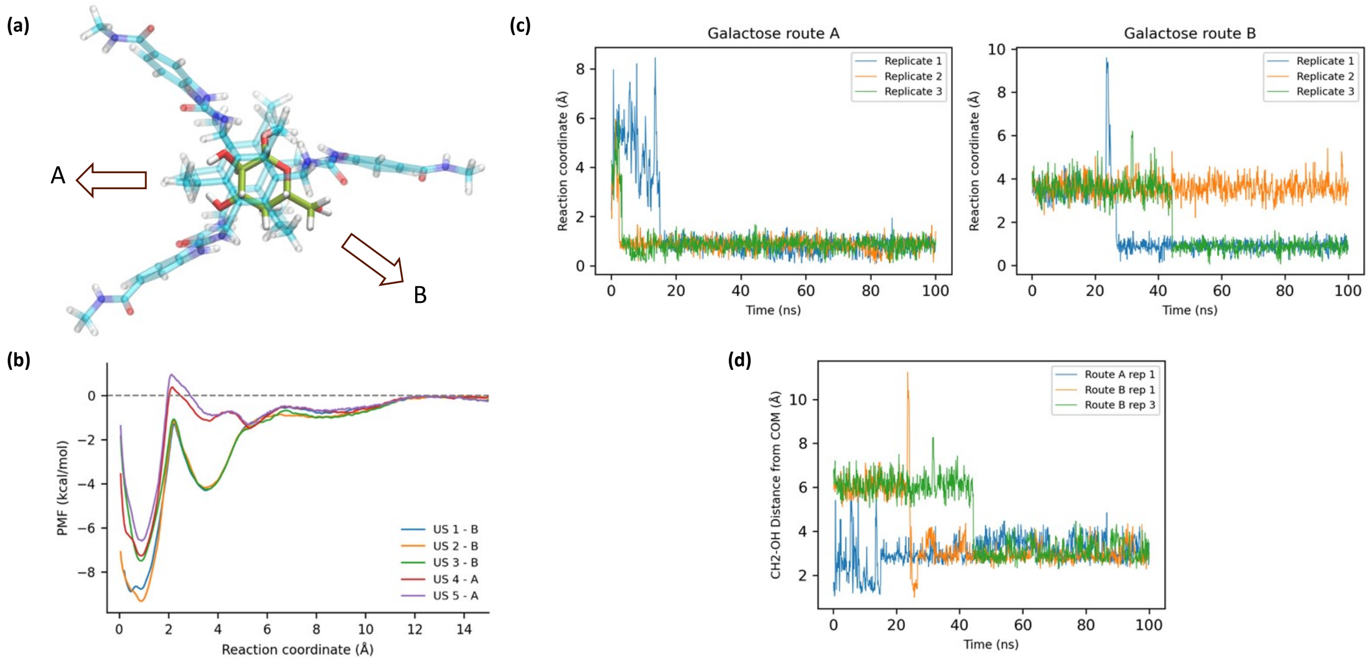

Figure S3. **Influence of exit direction on galactose PMF shape.** Steered molecular dynamics simulations revealed that the direction of sugar unbinding from the GluHUT cavity significantly affects the PMF, especially for galactose. (a) Two distinct unbinding paths, A and B, were identified for galactose. (b) Path B exhibited a pronounced PMF minimum around RC ≈ 4 Å. (c) To examine the stability of this minimum, three independent 100 ns unrestrained simulations were initiated from RC ≈ 4 Å for both paths. The time evolution of the reaction coordinate showed that complexes formed along path B were relatively long-lived with two replicas relaxed into the global minimum, while one remained near RC ≈ 4 Å. In contrast, complexes along path A were short-lived, with galactose consistently falling into a global minimum at RC ≈ 1 Å. (d) No interconversion between the two species was observed across replicates, as indicated by the COM distance between the cavity and the sugar's CH_2OH group, used here as a proxy for sugar rotation. In route B, galactose either re-entered the cavity directly without rotation (replicate 3) or moved further along the RC toward bulk solvent before returning via path A (replicate 1).

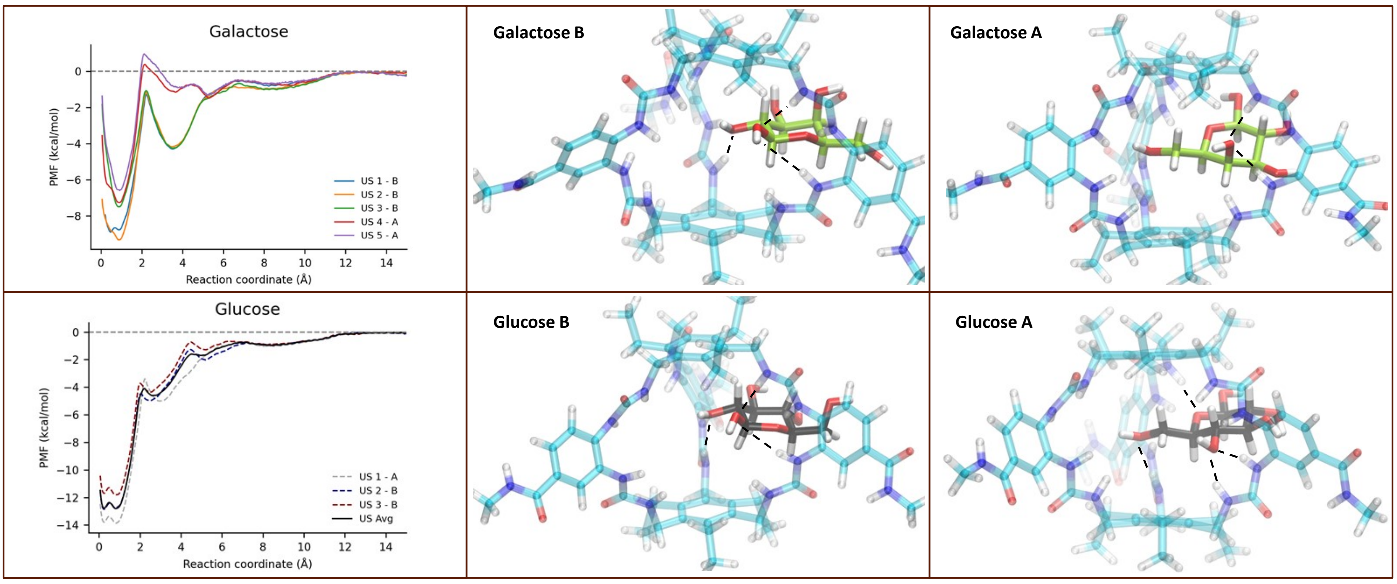

Figure S4. Exit direction influences galactose but not glucose. Both galactose path B and glucose paths A and B display a PMF minimum around RC ≈ 3-4 Å. While the unbinding route has little effect on the PMF shape for glucose, it strongly influences that of galactose. Representative snapshots at RC ≈ 4 Å illustrate the structural differences. In both glucose paths A and B, the sugar forms multiple hydrogen bonds with the receptor, adopting a relatively flat orientation. For galactose, however, path B features direct hydrogen bonding through the ring oxygens, whereas in path A the sugar undergoes significant rotation to form hydrogen bonds, driven by the steric hindrance of the bulky axial -OH group. This conformational constraint accounts for the absence of a pronounced minimum in galactose path A.

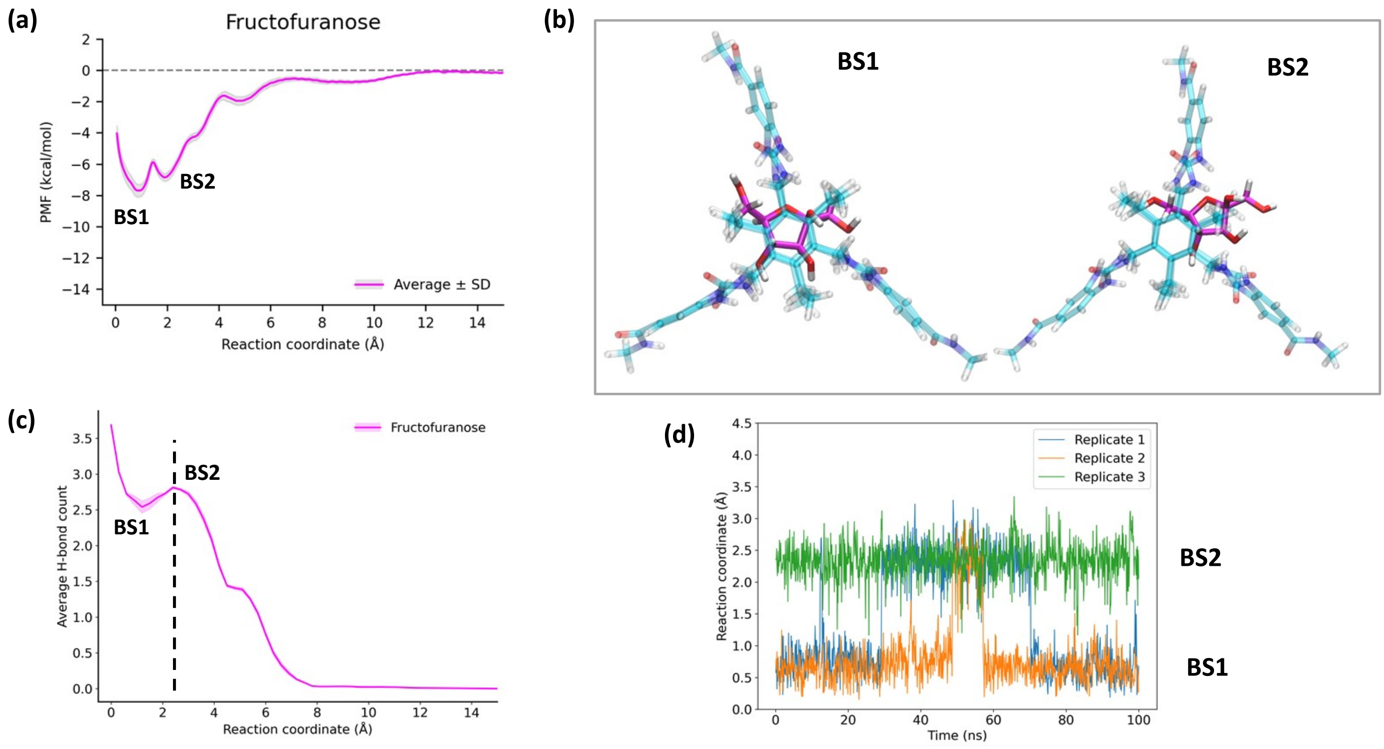

Figure S5. Fructofuranose has two bound states. (a) The PMF for fructofuranose reveals two bound states of comparable stability: BS1 at −7.9 kcal mol^{-1} and BS 2 at −7.0 kcal mol^{-1}. (b) Snapshots show BS1 adopting a central pose near RC ≈ 0.6 Å, while BS2 occupies an offset position at the cavity edge (RC ≈ 2.5 Å). (c) The GluHUT-fructofuranose hydrogen bonding is strongest at RC ≈ 2.5 Å. (d) Monitoring the reaction coordinate in three 100 ns unrestrained simulations demonstrates that the two bound states readily interconvert on the simulation timescale.

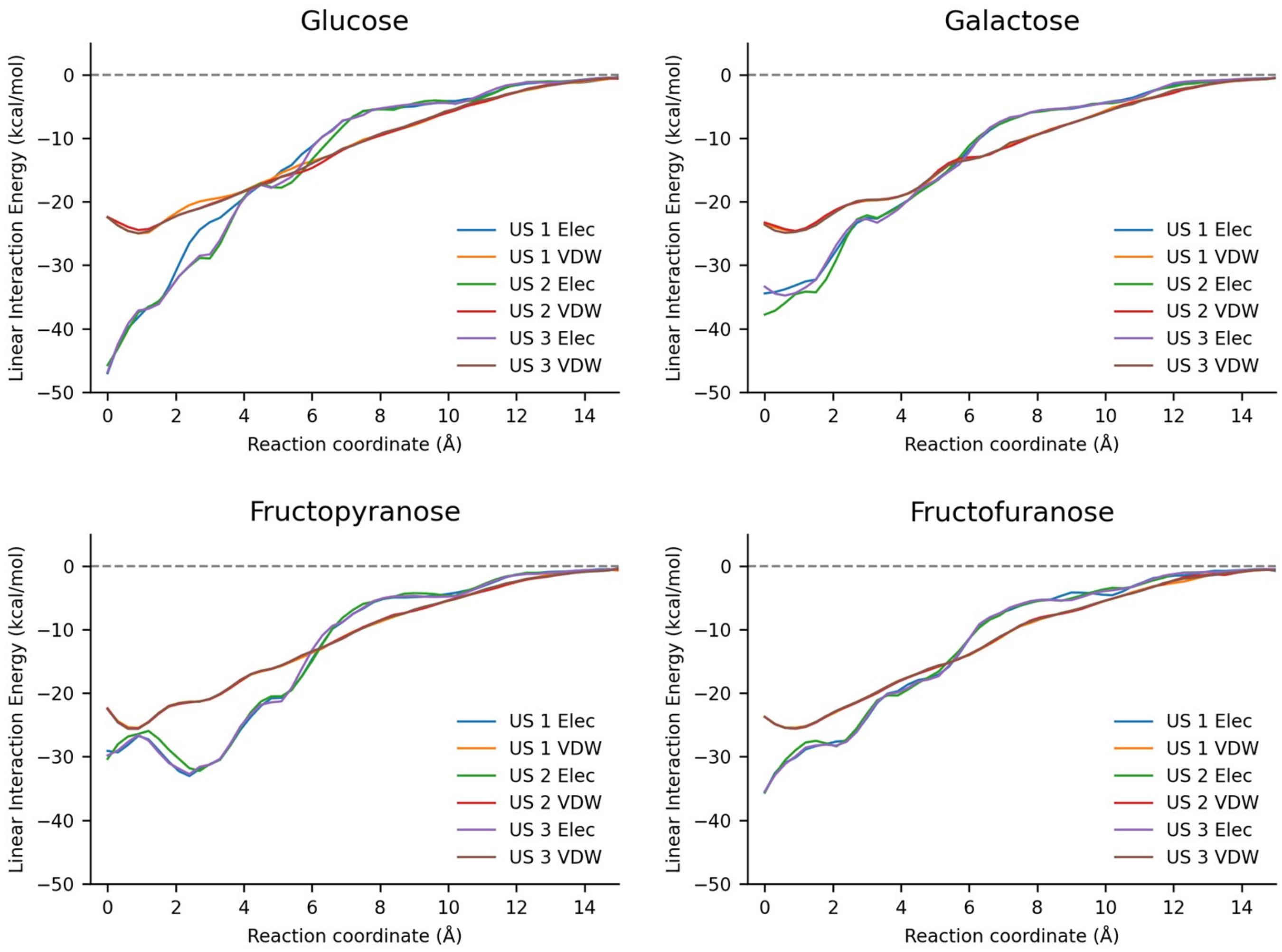

Figure S6. Interaction energies along the reaction coordinate. Linear interaction energy (LIE) analysis was performed for each umbrella sampling window and guest, decomposing the host-guest interactions into electrostatic (Elec) and van der Waals (VDW) components along the reaction coordinate.

Table S1. Water occupancy of the GluHUT cavity. Average number of water oxygen atoms within 2.65 Å of the urea -NH groups, computed for both the guest-bound and empty cavity states. The 2.65 Å cutoff corresponds to the first minimum of the radial distribution function (RDF).

Sugar	Water occupancy
Glucose	1.66 ± 0.01
Galactose	2.27 ± 0.01
Fructopyranose	2.73 ± 0.01
Fructofuranose	2.51 ± 0.01
Empty receptor	6.97 ± 0.04

Table S2. Hydrogen bonding between urea protons and water. Average number of hydrogen bonds formed between urea -NH groups and water oxygen atoms, with standard errors calculated from three independent replicates.

Sugar	Water – Urea H-bonds
Glucose	0.46 ± 0.009
Galactose	0.78 ± 0.010
Fructopyranose	0.97 ± 0.012
Fructofuranose	0.91 ± 0.012
Empty receptor	3.05 ± 0.027

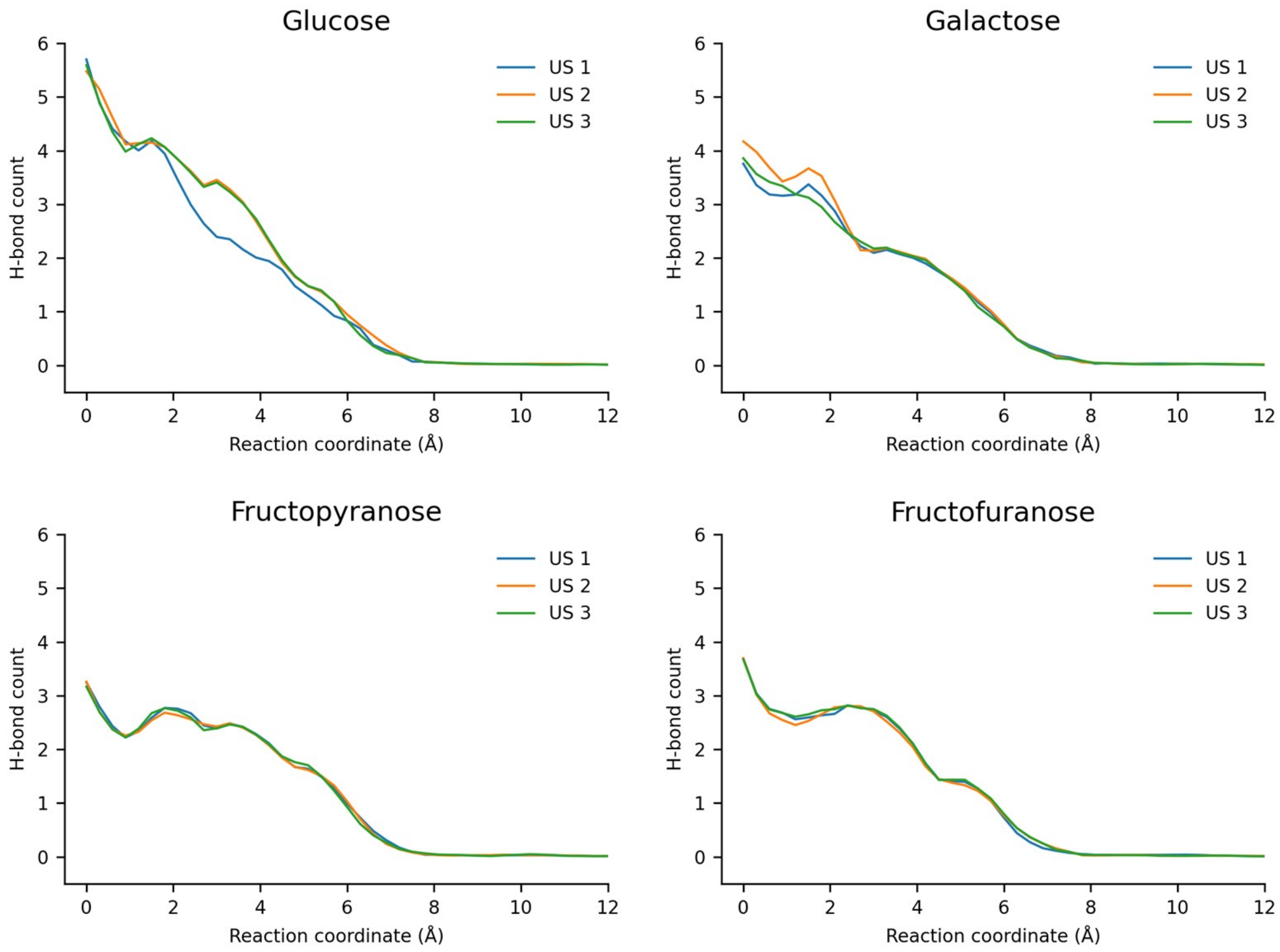

Figure S7. Hydrogen bonding between receptor and guest along the reaction coordinate. Hydrogen bonds formed between GluHUT and each of the four guest molecules were analysed from the fully bound state (RC = 0.0 Å) to the fully unbound state (RC = 12.0 Å), using the default *cpptraj* cutoff criteria. The guest molecules acted solely as hydrogen bond acceptors, with the urea -NH groups of GluHUT serving as the principal donors.

Table S3. Hydrogen bonding between each sugar and the receptor in the bound state. Total and per-oxygen hydrogen bond counts between each sugar and the receptor, calculated from three 100 ns unrestrained simulations. Standard errors of the mean (SEM) were determined from the average H-bond counts across the three replicates. For fructofuranose, three additional simulations were performed, and only complexes corresponding to the deepest free energy minimum (RC ≈ 0.6 Å) were included in the analysis.

Sugar	O1	O2	O3	O4	O5	O6	Total
Glucose	0.38 ± 0.008	1.10 ± 0.009	0.86 ± 0.008	0.85 ± 0.009	0.24 ± 0.006	0.69 ± 0.009	4.12 ± 0.009
Galactose	0.37 ± 0.007	0.87 ± 0.009	0.56 ± 0.008	0.29 ± 0.006	0.19 ± 0.005	0.85 ± 0.009	3.14 ± 0.007
Fructopyranose	0.56 ± 0.006	0.18 ± 0.005	0.53 ± 0.008	0.42 ± 0.007	0.34 ± 0.006	0.28 ± 0.006	2.32 ± 0.006
Fructofuranose	0.54 ± 0.007	0.24 ± 0.006	0.72± 0.009	0.87 ± 0.008	0.28 ± 0.006	0.56 ± 0.008	3.23 ± 0.007

Table S4. Definition of H-bonding scores. Scores were calculated as the mean hydrogen bond count across three simulations, divided by two (reflecting the maximum of two H-bonds per oxygen atom) and expressed as a percentage. See Fig. S8 for the oxygen naming scheme.

Sugar	O1	O2	O3	O4	O5	O6
Glucose	19	55	43	43	12	35
Galactose	19	44	28	15	10	43
Fructopyranose	28	9	27	21	17	14
Fructofuranose	27	12	36	44	14	28

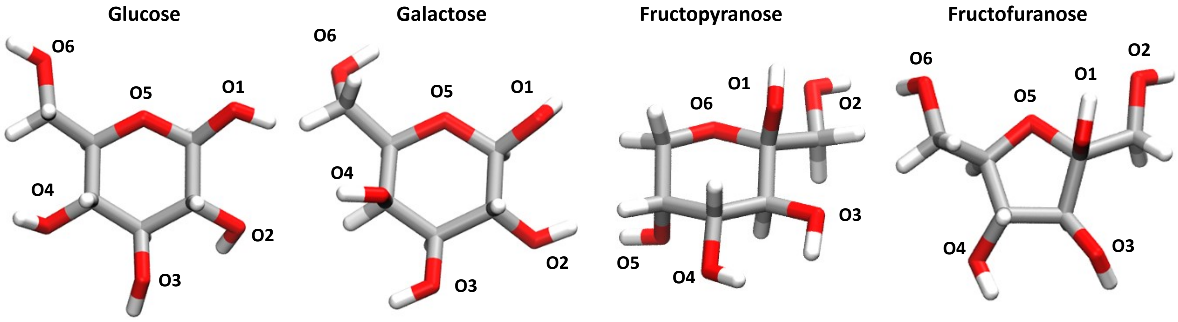

Figure S8. Structures of the sugars studied. Molecular structures of the four sugars investigated in this work, showing the labelled oxygen atoms used for hydrogen bonding analysis.

Table S5. Hydrogen bonding between guest hydroxyl groups and water. Average hydrogen bond counts between guest hydroxyl groups and surrounding water molecules, with -OH groups considered as both donors and acceptors. Standard errors of the mean (SEM) were calculated from three independent 100 ns unrestrained simulations. The O4 position in glucose and galactose is highlighted to illustrate their differing interactions with water, with glucose forming an average of 0.7 H-bonds compared to 0.1 for galactose.

Sugar	O1 - mean	O1 - SEM	O2 - mean	O2 - SEM	O3 - mean	O3 - SEM	O4 - mean	O4 - SEM	O5 - mean	O5 - SEM	O6 – mean	O6 - SEM	Total - mean	Total - SEM
Glucose - donor	0.836	0.005	0.747	0.006	0.703	0.006	0.718	0.006	0	0	0.682	0.006	3.85	0.014
Glucose - Acceptor	0.076	0.003	0.004	0.001	0.008	0.001	0.000	0.000	0.002	0.001	0.077	0.003		
Galactose - donor	0.797	0.005	0.714	0.006	0.651	0.006	0.143	0.005	0	0	0.630	0.006	3.19	0.014
Galactose - Acceptor	0.080	0.003	0.010	0.001	0.051	0.003	0.030	0.002	0	0	0.085	0.004		
Fructopyranose - donor	0.682	0.006	0.620	0.006	0.628	0.006	0.681	0.006	0.517	0.006	0	0	3.70	0.016
Fructopyranose - Acceptor	0.002	0.001	0.382	0.006	0.005	0.001	0.055	0.003	0.129	0.004	0	0		
Fructofuranose - donor	0.552	0.006	0.633	0.006	0.535	0.006	0.683	0.006	0	0	0.640	0.006	3.49	0.016
Fructofuranose - Acceptor	0.007	0.001	0.238	0.006	0.048	0.003	0.043	0.003	0	0	0.108	0.004		
